# Analysis of clinical characteristics and influencing factors of fever after electroconvulsive therapy: a retrospective study from the Chinese population

**DOI:** 10.3389/fpsyt.2025.1587179

**Published:** 2025-08-12

**Authors:** Mengmeng Zhang, Rui Xu, Juan Wang, Chunyan Wu, Huicong Ren, Zhaohui Zhang, Juan Li

**Affiliations:** ^1^ Psychiatry Department, The Second Affiliated Hospital of Xinxiang Medical University, Xinxiang, China; ^2^ Henan Collaborative Innovation Center of Prevention and Treatment of Mental Disorder, Xinxiang Medical University, Xinxiang, China; ^3^ Psychiatry Department, The First Affiliated Hospital of Xinxiang Medical University, Xinxiang, China; ^4^ Henan Engineering Research Center of Physical Diagnostics and Treatment Technology for the Mental and Neurological Disease,Xinxiang Medical University, Xinxiang, China; ^5^ Henan Key Laboratory of Neurorestoratology, Xinxiang Medical University, Xinxiang, China; ^6^ Xinxiang Key Laboratory of Child and Adolescent Psychiatry, The Second Affiliated Hospital of Xinxiang Medical University, Xinxiang, China

**Keywords:** electroconvulsive therapy (ECT), fever, mental disorders, risk factors, incidence

## Abstract

**Background:**

Although well-established as a first-line treatment for psychiatric disorders, electroconvulsive therapy (ECT) carries risks of adverse effects, including fever. The purpose of this study was to elucidate the incidence, clinical characteristics, and risk factors related to fever after ECT.

**Methods:**

We retrospectively analyzed medical records of patients receiving ECT at the Second Affiliated Hospital of Xinxiang Medical University (April 2019–January 2020). Patients were subsequently divided into two groups: a fever group, in which the body temperature was ≥ 38°C; and a non-fever group, in which the body temperature was <38°C.

**Results:**

A total of 895 patients underwent 7801 units of ECT treatment. Fever was analyzed at the patient and treatment unit level. At the patient level, 104 out of 895 patients (11.6%) experienced at least one fever within 24 hours after ECT, Compared with the non-fever group, the fever group showed statistically significant differences in age, gender, types of psychiatric ward (closed or open), and anesthetic type (all *P* < 0.05) but not in the total number of ECT units or diagnoses, Logistic regression analysis identified the risk variables for fever as younger age (≤ 29), closed psychiatric ward, etomidate administration, and being male; and at the treatment units level, among the 7,801 ECT units, fever occurred in 129 units (1.7%), with a median maximum temperature of 38.5 (38.0–40.3)°C. Following ECT, 55.8% (72/129) of the fever unit temperatures returned to normal body temperature as assessed by clinical observation or cooling measures, whereas 44.2% (57/129) required cooling combined with antibiotics. Compared to baseline, the fever units had higher white blood cell and neutrophil counts (*P* < 0.001) but lower lymphocyte counts (*P* < 0.001). In 79.8% (103/129) of the units, the fever was observed during the first 5–8 hours after the ECT treatment was completed, with 94.6% (122/129) of the units returning to normal body temperature within 24 hours of treatment. Only 5.4% (7/129) of the units opted to discontinue ECT treatment due to fever.

**Conclusion:**

We found that fever after ECT requires attention in clinical practice. Although the direct impact of fever after ECT treatment is limited, given its potential risks, it is advised to focus on strengthening the temperature monitoring of high-risk groups.

## 1 Introduction

Electroconvulsive therapy (ECT) is a method for treating severe mental disorders, which works by inducing a therapeutic seizure through a safe electrical stimulation of the brain. While pharmacotherapy remains the most frequently employed treatment for mental disorders, ECT is nevertheless recognized as a first-line therapeutic option in specific clinical scenarios (1), particularly in patients with depression accompanied by psychotic, suicidal, or treatment-resistant features (2). Indeed, in many cases, ECT can more effectively improve depressive symptoms compared to medication (3).

Modern ECT is performed under conditions of general anesthesia, muscle relaxants, and oxygen inhalation, and is usually well tolerated, although adverse reactions may still occur after treatment. Headaches and muscle pain are common adverse reactions after treatment, although the symptoms are usually mild (4). In addition, the incidence of nausea after treatment is also relatively high, which may be related to headaches and anesthetics (5). The most concerning side effect after ECT is cognitive function impairment, with common types including immediate postoperative confusion, attention and executive function issues, anterograde amnesia, and retrograde amnesia (6). Additionally, 1%–2% of patients may experience prolonged seizures (>180 s) (7), which increases the risk of consciousness impairment and memory decline.

The adverse reactions after ECT treatment have drawn significant attention, In clinical practice, we have observed that some patients experience fever after ECT; however, the number of studies is limited to a few case reports (8–10) and small retrospective studies (11). Overall, the reported incidence of fever after ECT varies significantly, ranging from 5.3% to 54.2% (12–16). Regarding the clinical features of fever, the degree and duration after ECT have been examined in several studies (13, 14). However, treatment measures and effects of fever after ECT and the impact of fever on ECT treatment course, among other clinical features, have not been reported. Additionally, literature shows inconsistent results in terms of the risk factors for developing fever after ECT (14, 16).

Considering that fever after ECT can cause subjective discomfort for patients and reduce treatment compliance, we believe that previous studies (11–16) have inadequately described the clinical characteristics of fever after ECT. Specifically, these studies have reported widely varying incidence rates and conflicting evidence on anesthetic-related risk factors. This gap necessitates further research. Here, we investigated the incidence, clinical characteristics, and risk factors of fever after ECT in Chinese psychiatric patients.

## 2 Methods

### 2.1 Study population

A retrospective study examined the medical records of patients underwent ECT treatment at the Second Affiliated Hospital of Xinxiang Medical University (Xinxiang, China) from April 2019 to January 2020. The demographic characteristics, clinical features, and anesthetic agents of all patients undergoing ECT were collected through the hospital’s electronic medical chart system. The study was approved by the hospital’s ethics committee (No. XYEFYLL-2023-30), and due to the retrospective design, no informed consent was required.

The inclusion criteria: 1) inpatients diagnosed with major mental disorders (ICD-10); and 2) received ECT treatment. Exclusion criteria: 1) fevers were due to infections (bacterial, COVID-19), other physical diseases, or medication; and 2) incomplete medical record; and 3) due to medication.

### 2.2 Study design

All enrolled patients were divided into a fever group and non-fever group based on whether they experienced fever within 24 h after ECT treatment course. As fever did not occur after every ECT session, we defined each individual ECT session as a ‘unit’ for analysis. In this study, we defined fever as an axillary temperature ≥ 38.0°C (17, 18). The fever group included patients with a temperature ≥ 38.0°C within 24 h after any ECT units.

**Figure 1:**
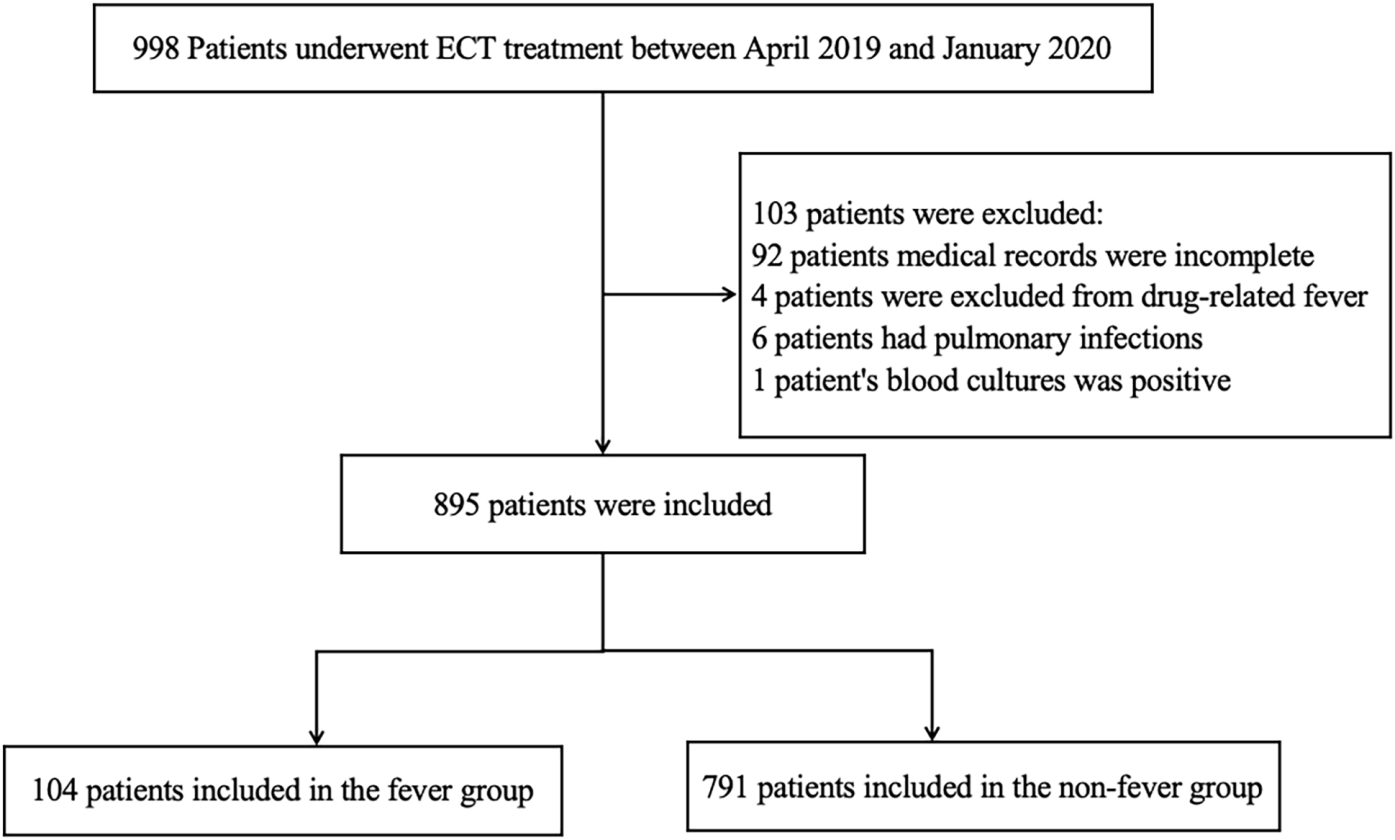
Flowchart of the study on fever after ECT treatment.

A total of 998 patients underwent ECT treatment between April 2019 and January 2020, of which 103 patients were ultimately excluded. The reasons for exclusion were 92 with incomplete data, four with drug allergies, six with pulmonary infections after fever, and one with infection. Ultimately, 895 patients were included in the study, as shown in **Figure 1**. The original 998 patients and the 895 included patients were evenly matched in terms of age, gender, and other clinical variables (including inpatient area and incidence of fever), suggesting that missing data did not affect the final results.

### 2.3 Prevalence of fever after ECT

The incidence of fever after ECT was calculated by dividing the number of patients who developed fever by the total number of enrolled patients; similarly, the incidence of fever per unit was determined by dividing the number of fever cases by the total number of units in the study. Considering that the physical development of adolescents is ongoing, the temperature regulation mechanisms of such patients are different from those of adults. Therefore, we specifically focused on the incidence of fever in adolescent patients after ECT.

### 2.4 ECT procedure and related parameters

Each patient underwent a comprehensive assessment and preparation prior to ECT, including blood routine tests, biochemistry tests, electrocardiogram, electroencephalogram, and chest computerized tomography. Patients received oxygen supplementation until spontaneous breathing resumed. Before anesthesia, 0.5–1.0 mg atropine was administered intravenously (IV). The anesthesiologist selected etomidate (0.15–0.30 mg/kg) or propofol (1.0–2.0 mg/kg) for anesthesia. Following loss of consciousness, 0.50–1.25 mg/kg succinylcholine was administered IV for muscle relaxation. Standard bi-temporal ECT was performed using the MECTA Spectrum 5000Q device (MECTA Corporation, Tualatin, OR, USA). The electrical parameters were as follows: pulse width 0.25 ms, frequency 60 Hz, duration 2 s, and current 0.9 A. In this study, an age-related method (%charge = patient’s age; 100% = 504 initial treatment charge dose) was used to estimate the initial treatment charge dose (19). Each ECT session was conducted three times a week, during the hours between 06:30 a.m. and 10:30 a.m. During the treatment, heart rate, blood oxygen saturation, and respiration were monitored.

### 2.5 Statistical analysis

Statistical analysis was performed using SPSSv26.0 (IBM Corp., Armonk, NY, USA). Categorical data are presented as numbers (%), and continuous data are presented as mean ± standard deviation. Continuous variables were analyzed using t-tests (normally distributed) or Mann-Whitney U tests (non-normal), while categorical variables used χ^2^ tests with results expressed as n (%). Variables showing intergroup differences underwent multivariate logistic regression to evaluate fever incidence impact. Statistical significance was defined as two-tailed *P* < 0.05.

## 3 Results

### 3.1 The incidence of fever after ECT

A total of 895 patients underwent 7801 units of ECT treatment. Fever was assessed using two distinct levels. At the patient level, 104 out of 895 patients (11.6%) experienced at least one fever within 24 hours after ECT. At the treatment unit level, among the 7,801 ECT units, fever occurred in 129 units (1.7%). We specifically focused on fever occurrence among adolescent patients (13–18 years old) after ECT and found that among patients under 18 years old, 26.1% (18/69, 95% CI: 14.4%–38.3%) experienced fever after ECT, while only 10.4% (86/826, 95% CI: 8.6%–12.9%) of patients aged 18 and older experienced fever after ECT (*P* < 0.001).

### 3.2 Clinical characteristics of patients between the fever group and non-fever group

**Table 1** presents the clinical characteristics of 895 patients receiving ECT. The median age of patients in the fever and non-fever groups was statistically different [24 (range: 14–54) vs 29 (range: 13–77); *P*< 0.05]. Fever incidence differed significantly by setting: 12.4% (97/780; 95% CI: 10.3%–14.9%) in closed wards versus 6.1% (7/115; 95% CI: 2.8%–12.3%) in open wards (*P* < 0.05). Gender differences were significant, with males showing a higher incidence (18.3% [65/356; 95% CI: 14.5%–22.6%]) than females (7.2% [39/539; 95% CI: 5.3%–9.7%]; *P* < 0.05). Anesthetic agent usage significantly affected outcomes: etomidate-associated fever (12.5%, 101/811; 95% CI: 10.3%–15.0%) exceeded propofol-associated cases (3.6%, 3/84; 95% CI: 1.2%–10.1%; *P* < 0.05). However no significant differences existed in total ECT treatments or diagnoses.

**Table 1:** Demographic and clinical characteristics between patients in the fever group and non-fever group, (*n* = 895).

Variables	Fever group (*n* = 104)	Non-fever group (*n* = 791)	Statistics	*P* value
Median age (years), [range]	24 (14–54)	29 (13–77)	z = −5.257	<0.0001*
Age (years), *n* (%)			*χ* ^2^ = 16.203	<0.0001*
≦29	74 (71.2%)	397 (50.2%)		
>29	30 (28.8%)	394 (49.8%)		
Age (years), *n* (%)			χ2 = 15.236	<0.0001*
<18	18 (26.1%)	86 (10.4%)		
≧18	51 (73.9%)	730 (89.6%)		
Gender, *n* (%)			*χ* ^2^ = 25.365	<0.0001*
Male	65 (62.5%)	291 (36.8%)		
Female	39 (37.5%)	500 (63.2%)		
Total number of ECT units for the same patient, [mean (± SD)]	9.14 (3.58)	8.67 (3.04)	*χ* ^2^ = 2.271	0.132
Diagnosis, *n* (%)			*χ* ^2^ = 2.747	0.432
Schizophrenia	60 (57.7%)	480 (60.7%)		
Depression	15 (14.4%)	120 (15.2%)		
Bipolar disorder	18 (17.3%)	92 (11.6%)		
Other mental disorders	11 (10.6%)	99 (12.5%)		
Inpatient area, *n* (%)				
Open ward	7 (6.7%)	108 (13.7%)	*χ* ^2^ = 4.06	0.044*
Closed ward	97 (93.3%)	683 (86.3%)		
Anesthetic, *n* (%)			*χ* ^2^ = 5.847	0.016*
Etomidate	101 (97.1%)	710 (89.8%)		
Propofol	3 (2.9%)	81 (10.2%)		

*Statistical significance at two-tailed *P* < 0.05.Bold values indicate p<0.05.

### 3.3 Comparison demographic and clinical characteristics between fever and non-fever Units

The specific details of the 129 fever units after ECT treatment are shown in **Table 2**. The median age was 23 years (14–54 years). Overall, 62.8% (81/129) of the fever units were male, 92.2% (119/129) came from closed wards, and 97.7% (126/129) were anesthetized using etomidate injection. A total of 792 units were anesthetized using propofol injection, three of which experienced fever.

**Table 2:** Comparison demographic and clinical characteristics between fever units and non-fever units (*n* = 7801).

Variables	Fever units (*n* = 129)	Non-fever units (*n* = 7672)	Stats	*P* value
Median age (years), [range]	23 (14–54)	29 (13–77)	z = −5.987	<0.0001*
Gender, *n* (%)			*χ* ^2^ = 25.728	<0.0001*
Male	81 (62.8%)	3118 (40.6%)		
Female	48 (71.2%)	4554 (59.4%)		
Inpatient area, *n* (%)				
Open ward	10 (7.8%)	828 (10.8%)	*χ* ^2^ = 1.223	**0.269**
Closed ward	119 (92.2%)	6844 (89.2%)		
Anesthetic, *n* (%)			*χ* ^2^ = 8.790	0.003*
Etomidate	126 (97.7%)	6884 (89.7%)		
Propofol	3 (2.3%)	788 (10.3%)		

*Statistical significance at two-tailed *P* < 0.05.Bold values indicate p<0.05.

### 3.4 Clinical characteristics of the fever units

#### 3.4.1 Frequency and temperature profile

The clinical characteristics of the fever units are shown in **Table 3**. Fever occurred once in 86 fever units (66.7%), twice in 14 (10.9%), three times in one (0.8%), and four times in three (2.3%), which resulted in a mean number of episodes per unit (N = 104) of 1.24 ± 0.62.

**Table 3:** Clinical characteristics of fever units after ECT treatment (*n* = 129).

Variables	Categories	Frequency (*n*)	Percentage (%)
Fever unit for the same patient	1	86	66.7
	2	14	10.9
	3	1	0.8
	4	3	2.3
Degree of fever	38.0°C ≦ T < 39.0°C	92	71.3
	39.0°C ≦ T < 40.0°C	37	28.5
	40.0°C ≦ T	2	0.02
Fever occurred after first ECT unit	Yes	40	31
	No	89	69
Fever period after ECT	1-4 h	14	10.9
	5-8 h	103	79.8
	9-24 h	12	9.3
Treatment measures	Clinical observation or cooling	72	55.8
	Cooling and antibiotic treatment	57	44.2
Duration of fever	t ≦ 24 h	122	94.6
	24 h < t ≦ 48 h	6	4.7
	48 h < t ≦ 72 h	1	0.8
Effects on the ECT	Continue treatment	114	88.4
	Prolonged treatment interval	8	6.2
	Stop treatment	7	5.2

T, temperature; t, time; h, hour.

#### 3.4.2 Temperature profile

The median peak body temperature across the fever units was 38.5°C (range: 38.0–40.3°C). The temperature distribution of the fever units was as follows: 71.3% (92/129) measured 38.0–38.9°C, 28.5% (35/129) 39.0–39.9°C, and 1.6% (2/129) ≥ 40.0°C.

#### 3.4.3 Temporal pattern

Overall, fever occurred in 31% of the fever units (40/129) after the first treatment session, with most cases (79.8%, 103/129) emerging within 5–8 hours after treatment, while in 10.9% (14/129) and 9.3% (13/129) of fever units the fever occurred within 1–4 and 9–24 hours, respectively.

#### 3.4.4 Recovery trajectory

All fever units achieved normothermia within 72 hours after treatment, predominantly via clinical observation/cooling (55.8%, 72/129) or antibiotics (44.2%, 57/129). Recovery to normothermia occurred in 94.6% (122/129) of fever units within 24 hours after treatment, whereas in 4.7% (6/129) and 0.8% (1/129) fever units, it occurred within 24–48 and 48–72 hours, respectively. No significant difference was found between the 24 hour-recovery rate between antibiotic-treated (96.5% [55/57], 95% CI: 88.1%–99.0%) and non-antibiotic patients (93.1% [67/72], 95% CI: 84.8%–97.0%; P = 0.463, RR = 1.04, 95% CI:0.95–1.13).

#### 3.4.5 Treatment continuation

Following fever, 88.4% (114/129) of units continued ECT treatments as usual, whereas 6.2% (8/129) and 5.4% (7/129) either extended treatment intervals or discontinued therapy, respectively.

#### 3.4.6 Inflammatory dynamics

Among 129 the fever units, 98 underwent paired pre/post-ECT blood tests. Results indicated a significantly increased white blood cell (11.78 ± 3.62 vs baseline 6.81 ± 6.09 × 10^9^/L; *P* < 0.001, *t* = 13.461) and neutrophil count (9.72 ± 3.47 vs 4.28 ± 1.94 × 10^9^/L; *P* < 0.001, *t* = 14.673), with a significant reduction in lymphocytes (1.38 ± 0.47 vs 1.89 ± 0.63 × 10^9^/L; *P* < 0.001, *t* = −7.548). Complete hematologic shifts are detailed in **Table 4**.

**Table 4:** Blood routine of 98 fever units before and after ECT.

Variables	Pre-ECT	After ECT	Statistics	Df	*P* value
WBC count, ×10^9^/L	6.81 (2.09)	11.78 (3.62)	t = 13.461	97	<0.0001*
NEUT count, ×10^9^/L	4.28 (1.94)	9.72 (3.47)	t = 14.673	97	<0.0001*
LYMPH count, ×10^9^/L	1.89 (0.63)	1.38 (0.48)	t = −7.548	97	<0.0001*

*Statistical significance at two-tailed *P* < 0.05.Bold values indicate p<0.05.

### 3.5 Analysis of risk factors for fever after ECT

Logistic regression analysis was performed with collinearity diagnostics to confirm all variance inflation factor values < 5. The Hosmer-Lemeshow test indicated an adequate model fit (χ² = 2.383, df = 5, *P* = 0.794). Age (OR = 0.435, *P* < 0.001), gender (OR = 0.386, *P* < 0.001), inpatient ward type (OR = 2.802, *P* = 0.016), and anesthetic agent (OR = 3.856, *P* = 0.026) were significantly associated with fever. The total ECT sessions and diagnosis showed no independent correlation. Detailed results are shown in **Table 5**.

**Table 5:** Multivariable logistic regression analysis of factors associated with fever after ECT treatment (*n* = 895).

Variables	VIF	OR	95% CI	*P* values
Age	1.050	0.435	0.273–0.692	<0.001*
Gender	1.046	0.386	0.250–0.597	<0.001*
Diagnosis	1.027	1.605	0.702–3.670	0.262
Type of inpatient ward	1.045	2.802	1.213–6.457	0.016*
Anesthetic	1.011	3.856	1.176–12.641	0.026*
Total number of ECT units	1.060	1.059	0.678–1.655	0.801

OR, adjusted odds ratio; CI, confidence interval; *statistical significance at two-tailed *P* < 0.05; VIF, variance inflation factor.

## 4 Discussion

To the best of our knowledge, this study includes the largest sample size (n = 895) investigating the incidence of fever after ECT. The findings demonstrate that fever is common, with an elevated risk among males, adolescents, patients in closed psychiatric wards, and those given etomidate as an anesthetic. We speculate that the elevated risk among males may be associated with two mechanisms: (1) increased heat production from larger skeletal muscle mass during ECT-induced contractions; and (2) sex-based immune modulation–testosterone-mediated suppression versus estrogen-enhanced responses in females (20). Minors and adolescents also showed an increased likelihood of fever, which is likely attributed to underdeveloped thermoregulatory mechanisms relative to adults (21).The association between closed psychiatric wards and fever, the first identified in the Chinese population, may arise from shared living conditions with poor air circulation that heightens exposure to respiratory pathogens, which is compounded by severe mental illnesses and reduced self-care capacities.

In agreement with previous findings (14), we found that, compared with propofol, etomidate usage during ECT was associated with an increased risk of fever. This increased risk may involve the adrenal suppression and prolonged seizure activity that are associated with etomidate. Specifically, etomidate is known to work on the hypothalamus to inhibit the secretion of adrenal cortical hormones (22), whereas not such effects are associated with propofol (23, 24). Indeed, a single IV dose of etomidate was found to significantly reduce cortisol levels and increase those of adrenocorticotropic hormone (25, 26). Notably, significant adrenal suppression was found to last for 6 to 8 hours after IV etomidate (23, 26, 27), but resolved within 12 to 24 hours (23, 28, 29). Additionally, adrenal insufficiency is known to be one cause of unexplained fever (30). Our findings indicated that the onset of fever occurred within 5–8 hours after ECT in 79.8% of the fever units, which coincided with the alterations observed during the cortisol suppression found in previous studies. In addition, studies have indicated that etomidate is linked to longer seizure activity than propofol (31, 32), and this generalized epileptic activity can raise body temperature (33). The length of seizures is also significant predictor of fever after ECT (11). Therefore, since etomidate is associated with longer seizure activity, it may have contributed to an increase in body temperature and resultant fever.

Our research further indicated that fever after ECT most commonly occurred during the afternoon of the day that the treatment was completed. Overall, the fevers were mild to moderate and transitory, with body temperature returning to normal within 24 hours. These clinical characteristics may explain why fever after ECT has often been overlooked. Therefore, we recommend monitoring for fever on the day of treatment completion, especially during the afternoon hours. If fever occurs after initial treatment, subsequent ECT treatments may induce fever.

Fever is usually seen as a sign of infection or systemic inflammation and can indicate serious or life-threatening diseases. We found that not all fevers after ECT were indicative of infection. Specifically, more than half of patient temperatures returned to normal body temperature through clinical observation or cooling measures. Interestingly, cooling combined with antimicrobial treatments was as effective as cooling or observation alone. Regardless, most patients’ temperatures returned to normal body temperature within 24 hour after fever onset. Given that the ECT treatment frequency in the current study was 2–3 times per week, fever did not impact the overall treatment course, and 94.6% (122/129) of fever units chose to continue or extend treatment interval, while only 5.4% (7/129) opted to discontinue ECT after experiencing fever. The 94.6% continuation rate after ECT shows that transient fever seldom requires changes to treatment protocols. This clinically significant finding suggests that in cases of fever without evidence of infection, prioritizing observation or physical cooling may be necessary. If the drop in body temperature is minimal, consider administering antibiotics, as this may help reduce unnecessary antibiotic use, which occurred in 44.2% of fever units, potentially lowering healthcare costs.

It is worth noting that this study has several limitations. First, due the retrospective study design, complete blood counts, C-reactive protein levels, and chest computed tomography scans were not performed after each fever occurrence; thus it is impossible to determine if the fevers were caused by infection. Second, the selection of etomidate and propofol was not completely randomized and the cohort size using propofol as an anesthetic was relatively small, which may have led to selection bias. However, we treated each ECT session as an independent unit to compare the fever rates after using two anesthetics; therefore this limitation did not affect the finding that etomidate administration was more likely to cause fever after ECT than propofol. Third, fever may be related to factors such as anesthesia time and duration of seizures. We did not measure adrenocorticotropic hormone or cortisol levels, nor did we compare the seizure time between etomidate and propofol. These factors represent important limitations of our research, and future studies should directly investigate the relationship between fever and neuroendocrine changes after ECT, as well as the relationship between seizure time and fever. Fourth, this study did not analyze the use of antipsychotic medications. Indeed, reports have shown that antipsychotic drugs can affect body temperature, with fluphenazine, olanzapine, and risperidone potentially lowering the axillary temperature in patients with psychosis (34), which we believe may lead to an underestimation of the incidence of fever after ECT. Finally, fevers have been associated with concomitant medications, comorbidities, body-mass index, hydration status, smoking, position of the electrodes, and other clinical factors parameters not addressed in the study. In the future, we will conduct prospective randomized controlled studies of fever after ECT.

## 5 Conclusions

This study found that fever after ECT requires attention in clinical practice. Although the direct impact of fever on the ECT treatment is relatively limited, given its potential risks, it is necessary to focus on strengthening the temperature monitoring of high-risk groups. To further clarify the relevant mechanisms and clinical significance, prospective studies should be conducted to further explore the occurrence mechanism of fever and its specific impact on treatment outcomes.

## Data Availability

The original contributions presented in the study are included in the article/supplementary material. Further inquiries can be directed to the corresponding authors.

## References

[1] Kellner CH Obbels J Sienaert P. When to consider electroconvulsive therapy (ECT). Acta Psychiatr Scand. (2020) 141:304–15. doi: 10.1111/acps.13134, PMID: 31774547

[2] Milev RV Giacobbe P Kennedy SH Blumberger DM Daskalakis ZJ Downar J. Canadian network for mood and anxiety treatments (CANMAT) 2016 clinical guidelines for the management of adults with major depressive disorder: section 4. Neurostimulation Treatments. Can J Psychiatry. (2016) 61:561–75. doi: 10.1177/0706743716660033, PMID: 27486154 PMC4994792

[3] The UK ECT Review Group. Efficacy and safety of electroconvulsive therapy in depressive disorders: a systematic review and meta-analysis. Lancet. (2003) 361:799–808. doi: 10.1016/S0140-6736(03)12705-5, PMID: 12642045

[4] Dinwiddie SH Huo D Gottlieb O. The course of myalgia and headache after electroconvulsive therapy. J ECT. (2010) 26:116–20. doi: 10.1097/YCT.0b013e3181b07c0a, PMID: 19710619

[5] Andrade C Arumugham SS Thirthalli J. Adverse effects of electroconvulsive therapy. Psychiatr Clin North Am. (2016) 39:513–30. doi: 10.1016/j.psc.2016.04.004, PMID: 27514303

[6] Kellner CH. Handbook of ECT: a guide to electroconvulsive therapy for practitioners. Cambridge, UK; New York, NY: Cambridge University Press (2019).

[7] Whittaker R Scott A Gardner M. The prevalence of prolonged cerebral seizures at the first treatment in a course of electroconvulsive therapy. J ECT. (2007) 23:11–3. doi: 10.1097/01.yct.0000263253.14044.3a, PMID: 17435565

[8] Belson C Register S Bedford J. Transient febrile episodes after electroconvulsive therapy (ECT). J ECT. (2021) 37:e26–7. doi: 10.1097/YCT.0000000000000751, PMID: 33625178

[9] Bryson EO Pasculli RM Briggs MC Popeo D Aloysi AS Kellner CH. Febrile reaction with elevated CPK after a single electroconvulsive therapy (ECT) in an adolescent patient with severe bipolar disorder. J ECT. (2012) 28:70–1. doi: 10.1097/YCT.0b013e31823dfeb0, PMID: 22343589

[10] Antosik-Wojcinska A Z Bzinkowska D Swiecicki L Bienkowski P. Post-ECT hyperthermia and rapid mood improvements: a case report. J Neuropsychiatry Clin Neurosci. (2014) 26:E21. doi: 10.1176/appi.neuropsych.13020042, PMID: 24763777

[11] Jo YT Lee J Joo YH. Fever as a side effect after electroconvulsive therapy. Neuropsychobiology. (2022) 81:19–27. doi: 10.1159/000511542, PMID: 34233323

[12] Xiao A Liang Q Shuai S Chen M. Observations on the adverse effects of modified electroconvulsive therapy in psychiatric patients (in chinese). J Nurs Sci. (2001) 16:485–6. doi: 10.3969/j.issn.1001-4152.2001.08.018

[13] Xie QF Ye B Chen H Wu WY. An analysis of fever in patients after modified electroconvulsive therapy (in chinese). J Jinggangshan Univ (Science Technology). (2009) 30:105–6. doi: 10.3969/j.issn.1674-8085.2009.02.037

[14] Li SS Deng P Li Y Luo WX Zhang QH. Comparison of fever after conventional and modified electroconvulsive therapy. Military Med J South China. (2014) 28:283–4. doi: 10.3969/j.issn.1009-2595.2014.03.031

[15] Li CY Li YY. Analysis of fever status and influencing factors after electroconvulsive therapy in hospitalized male psychiatric patients. Med Res Educ. (2018) 35:26–9. doi: 10.3969/j.issn.1674-490X.2018.03.004

[16] Deng CJ Yang JW Liu ZZ Ning T Nie S Huang X. Risk factors for electroconvulsive therapy-induced fever: a retrospective case-control study. Front Psychiatry. (2025) 15:1530533. doi: 10.3389/fpsyt.2024.1530533, PMID: 39925704 PMC11802507

[17] Liang HH Zhang MX Wen YM Xu XL Mao Z She YJ. The incidence of and risk factors for postoperative fever after cleft repair surgery in children. J Pediatr Nurs. (2019) 45:e89–94. doi: 10.1016/j.pedn.2019.01.009, PMID: 30738633

[18] Hayashi N Murai H Ishihara S Kitamura T Miki T Miwa T. Nationwide investigation of the current status of therapeutic neuroendoscopy for ventricular and paraventricular tumors in Japan. J Neurosurg. (2011) 115:1147–57. doi: 10.3171/2011.7.JNS101976, PMID: 21838511

[19] Mankad MV Beyer JL Weiner RD Krystal A. Clinical manual of electroconvulsive therapy. Arlington: American Psychiatric Publishing (2010).

[20] Yalcinkaya A Yalcinkaya R Sardh F Landegren N. Immune dynamics throughout life in relation to sex hormones and perspectives gained from gender-affirming hormone therapy. Front Immunol. (2024) 15:1501364. doi: 10.3389/fimmu.2024.1501364, PMID: 39885993 PMC11779622

[21] Cowgill LW Eleazer CD Auerbach BM Temple DH Okazaki K. Developmental variation in ecogeographic body proportions. Am J Phys Anthropol. (2012) 148:557–70. doi: 10.1002/ajpa.22072, PMID: 22623278

[22] Nicholson G Hall G. Hypothalamic–pituitary–adrenal function: anaesthetic implications. Anaest Intens Care Med. (2014) 15:473–6. doi: 10.1016/j.mpaic.2014.07.009

[23] Zhang Y Luo A An G Huang Y. Effect of propofol and etomidate for anesthesia induction on plasma total cortisol concentration. Zhongguo Yi Xue Ke Xue Yuan Xue Bao. (2000) 22:284–6. doi: 10.1007/BF02983513, PMID: 12903479

[24] Murakawa T Tsubo T Kudo T Kudo M Matsuki A. Effect of propofol as an agent for anesthetic induction on pituitary-adrenocortical function during anesthesia and surgery. Masui. (1998) 4711:1350–7. doi: 10.1007/BF03006885, PMID: 9852700

[25] Allolio B Stuttmann R Leonhard U Fischer H Winkelmann W. Adrenocortical suppression by a single induction dose of etomidate. Klin Wochenschr. (1984) 62:1014–7. doi: 10.1007/BF01711723, PMID: 6096626

[26] Fragen RJ Shanks CA Molteni A Avram MJ. Effects of etomidate on hormonal responses to surgical stress. Anesthesiology. (1984) 61:652–6. doi: 10.1097/00000542-198412000-00004, PMID: 6095701

[27] Wanscher M Tønnesen E Hüttel M Larsen K. Etomidate infusion and adrenocortical function: A study in elective surgery. Acta Anaesthesiologica Scandinavica. (1985) 29:483–85. doi: 10.1111/j.1399-6576.1985.tb02238.x, PMID: 4036533

[28] Hildreth AN Mejia VA Maxwell RA Smith PW Dart BW Barker DE. Adrenal suppression following a single dose of etomidate for rapid sequence induction: a prospective randomized study. J Trauma. (2008) 65:573–9. doi: 10.1097/TA.0b013e31818255e8, PMID: 18784570

[29] Schenarts CL Burton JH Riker RR. Adrenocortical dysfunction following etomidate induction in emergency department patients. Acad Emergency Med. (2001) 8:1–7. doi: 10.1111/j.1553-2712.2001.tb00537.x, PMID: 11136139

[30] Ko JW Lee SE Park JH Kim B. Risk factors that are associated with adrenal insufficiency among patients with fever of unknown origin. Postgraduate Med. (2023) 135:734–40. doi: 10.1080/00325481.2023.2261355, PMID: 37725479

[31] Hoyer C Kranaster L Janke C Sartorius A. Impact of the anesthetic agents ketamine, etomidate, thiopental, and propofol on seizure parameters and seizure quality in electroconvulsive therapy: a retrospective study. Eur Arch Psychiatry Clin Neurosci. (2014) 264:255–61. doi: 10.1007/s00406-013-0420-5, PMID: 23835527

[32] Singh PM Arora S Borle A Varma P Trikha A Goudra BG. Evaluation of etomidate for seizure duration in electroconvulsive therapy: a systematic review and meta-analysis. J ECT. (2015) 31:213–25. doi: 10.1097/YCT.0000000000000212, PMID: 25634566

[33] Wachtel TJ Steele GH Day JA. Natural history of fever following seizure. Arch Intern Med. (1987) 147:1153–5. doi: 10.1001/archinte.1987.00370060149024, PMID: 3592881

[34] Heh W Herrera J DeMet E Potkin S Costa J Sramek J. Neuroleptic-induced hypothermia associated with amelioration of psychosis inschizophrenia. Neuropsychopharmacology. (1988) 1:149–56. doi: 10.1016/0893-133x(88)90006-1, PMID: 3251495

